# Polymorphisms in *ADH1B* and *ALDH2* genes associated with the increased risk of gastric cancer in West Bengal, India

**DOI:** 10.1186/s12885-017-3713-7

**Published:** 2017-11-22

**Authors:** Sudakshina Ghosh, Biswabandhu Bankura, Soumee Ghosh, Makhan Lal Saha, Arup Kumar Pattanayak, Souvik Ghatak, Manalee Guha, Senthil Kumar Nachimuthu, Chinmoy Kumar Panda, Suvendu Maji, Subrata Chakraborty, Biswanath Maity, Madhusudan Das

**Affiliations:** 1 Department of Zoology, University of Calcutta, 35 Ballygunge Circular Road, Kolkata, West Bengal 700019 India; 2 Department of Biotechnology, Mizoram University, Tanhril, P.O Box No. 190, Aizawl, Mizoram India; 3 Department of Surgery, Institute of Post Graduate Medical Education & Research, 244 A.J.C Bose Road, Kolkata, West Bengal 700 020 India; 4 Department of Oncogene Regulation and Viral Associated Human Cancer, Chittaranjan National Cancer Institute, 37, S. P. Mukherjee Road, Kolkata, West Bengal 700026 India

**Keywords:** Gastric cancer, ADH1A, ADH1B, ADH1C, ALDH2

## Abstract

**Background:**

Gastric cancer (GC) is one of the most frequently diagnosed digestive tract cancers and carries a high risk of mortality. Acetaldehyde (AA), a carcinogenic intermediate of ethanol metabolism contributes to the risk of GC. The accumulation of AA largely depends on the activity of the major metabolic enzymes, alcohol dehydrogenase and aldehyde dehydrogenase encoded by the ADH (ADH1 gene cluster: ADH1A, ADH1B and ADH1C) and ALDH2 genes, respectively. This study aimed to evaluate the association between genetic variants in these genes and GC risk in West Bengal, India.

**Methods:**

We enrolled 105 GC patients (cases), and their corresponding sex, age and ethnicity was matched to 108 normal individuals (controls). Genotyping for ADH1A (rs1230025), ADH1B (rs3811802, rs1229982, rs1229984, rs6413413, rs4147536, rs2066702 and rs17033), ADH1C (rs698) and ALDH2 (rs886205, rs968529, rs16941667 and rs671) was performed using DNA sequencing and RFLP.

**Results:**

Genotype and allele frequency analysis of these SNPs revealed that G allele of rs17033 is a risk allele (A vs G: OR = 3.67, 95% CI = 1.54–8.75, *p* = 0.002) for GC. Significant association was also observed between rs671 and incidence of GC (*p* = 0.003). Moreover, smokers having the Lys allele of rs671 had a 7-fold increased risk of acquiring the disease (OR = 7.58, 95% CI = 1.34–42.78, *p* = 0.009).

**Conclusion:**

In conclusion, rs17033 of ADH1B and rs671 of ALDH2 SNPs were associated with GC risk and smoking habit may further modify the effect of rs671. Conversely, rs4147536 of ADH1B might have a protective role in our study population. Additional studies with a larger patient population are needed to confirm our results.

**Electronic supplementary material:**

The online version of this article (10.1186/s12885-017-3713-7) contains supplementary material, which is available to authorized users.

## Background

Gastric cancer (GC) is one of the most frequently diagnosed digestive tract cancers. The asymptomatic disease presentation with nonspecific signs and symptoms in its early stage results in relatively poor prognosis due to advanced disease progression and a high mortality rate [1, 2]. It is the fourth most common cancer and the third leading cause of global cancer death despite its declining incidence in the recent decade [3]. Worldwide it causes approximately 700,000 deaths each year [4]. In India, the prevalence of GC is low compared to that in western countries with the number of new GC cases numbering around 34,000 per annum. Male patients predominate with GC exhibiting a 2:1 male bias [5].In India, a wide variation is observed in the incidence of this disease, having four times higher rate in Southern India compared to the North [6, 7].The highest prevalence of GC has been documented from Mizoram, a North-Eastern state of India [8]. Though several types of cancer can occur in the stomach, adenocarcinomas are the most frequently diagnosed (90–95% of cases). It is well established that infection with *Helicobacter pylori* may predispose an individual to GC, but smoking, alcohol, diet, genetics and epigenetic factors may also contribute to disease risk [9–13]. In particular, a family history of cancer, especially stomach cancer, significantly increases the risk of deaths [14].

In 2007, the International Agency for Research on Cancer classified alcohol, which erodes the mucosal lining of the stomach, as a group 1 human carcinogen. Alcohol metabolism is mainly mediated by two classes of enzymes: alcohol dehydrogenases and aldehyde dehydrogenases. Although the liver is the major site of their expression, these enzymes are also found in the gastrointestinal (GI) tract [15]. In the GI tract, mucosal and/or bacterial alcohol dehydrogenases can produce acetaldehyde (AA) from ethanol. AA, a highly toxic intermediate, has direct mutagenic and carcinogenic effects by interfering DNA synthesis and repair [16]. Genetic variations in alcohol-metabolizing enzymes contribute to individual differences in ethanol metabolism that may increase the risk of ethanol associated pathologies. Individuals with enzyme variants that lead to either increased AA generation or failure of AA detoxification have been shown to have an increased cancer risk [17]. Recent evidence suggests that AA, as opposed to ethanol itself is responsible for the carcinogenic properties of alcohol [18]. Due to the critical function of alcohol and aldehyde dehydrogenases in controlling the conversion of alcohol to toxic intermediates, understanding how genetic variants in these genes contribute to GC development could provide new understanding into the role of alcohol consumption in encoding GC risk.

The *ADH1* gene cluster (*ADH1A*, *ADH1B* and *ADH1C*), responsible for the bulk of ethanol metabolism in the liver, is located on chromosome 4q23 [19]. Earlier reports revealed a significant association between a common 3’UTR flanking SNP near *ADH1A* (rs1230025) and GC risk. This association is further modified by alcohol intake [20]. Recent genome-wide association studies identified the variation of *ADH1B* rs1229984 as risk factor for esophageal cancer in a Japanese population. It has been postulated that individuals expressing *ADH1B* variants, in particular, could have altered rates of alcohol elimination [21].However, difference in ethnicity and gender along with variation in enzyme activity can modify carcinogenic potential [22]. Recent evidence from 35 case–control studies indicate that *ADH1C* Ile350Val (rs698) polymorphism may also contribute to cancer risk among Africans and Asians [23]. The *ALDH2* (mitochondrial aldehyde dehydrogenase) gene is located on chromosome 12q24.2. It is expressed in both liver and stomach and plays the major role for converting AA into nontoxic acetate [24–26]. Genetic polymorphisms in this gene modulate individual differences in AA accumulation. Single nucleotide polymorphisms (SNPs) of *ALDH2* gene can lead to structural and functional changes in the enzymes that could influence AA levels and, as a result may predispose people to GC. An earlier study has shown that *ALDH2* Glu504Lys (rs671) polymorphism interacts with alcohol drinking in determining stomach cancer risk [27]. However, findings have been inconsistent with regard to the association of *ADH1A, ADH1B, ADH1C* and *ALDH2* genes polymorphisms with GC risk. Also, to the best of our knowledge till date, no data of these genes with regard to GC has been reported from India. Thus, the present study was aimed to investigate the possible association of these genes polymorphisms with GC risk in a patient population from the state of West Bengal, India. Our results indicate that rs17033 and rs671 of *ADH1B* and *ALDH2* genes respectively were significantly associated with GC risk whereas rs4147536 of *ADH1B* might have a protective role in the study population.

## Methods

This study was approved by the institutional ethics committee of Institute of Post Graduate Medical Education & Research (IPGME & R), Kolkata, West Bengal, India. A signed informed consent was taken from each participant.

### Study subjects

Recruitment of 105 cases was accomplished in the Department of Surgery, IPGME & R, Kolkata, West Bengal, India from December 1, 2012 to April 30, 2015. All the subjects enrolled in our study were Bengali. Eligible cases included patients newly diagnosed and histopathologically confirmed gastric adenocarcinoma without any chronic disease. They were all unrelated patients diagnosed at a locally advanced stage of gastric cancer that required surgery. Histological gradations of tumour tissues were done based on the classification derived by Lauren (1965) [28]. One hundred and eight age, sex and ethnicity matched healthy control subjects were selected from the same geographical region and socioeconomic status with no cancer and familial history of neoplasms. Non-cancer status was confirmed by medical examinations, including radiographic examinations.

### Data collection

Each study participant was interviewed for their socio-demographic characteristic, life style, family history of cancer or other chronic diseases, smoking, drinking and dietary habits and physical activity (Additional file 1: Data S1).

### Genotyping of *ADH1A, ADH1B, ADH1C* and *ALDH2* polymorphisms

Genomic DNA was extracted from the peripheral blood collected from each of the participants. Genotyping for *ADH1A* (rs1230025), *ADH1B* (rs3811802, rs1229982, rs1229984, rs6413413, rs4147536, rs2066702, rs17033*),* and *ALDH2* (rs886205, rs968529, rs16941667*)* polymorphisms were performed using sequence of each of the specific fragment of genomic DNA. Specific primers were used to amplify each polymorphic DNA sequence by polymerase chain reaction (PCR) (Additional file 2: Table S1). PCR amplification was undertaken in a 30 μl volume containing 100 ng of DNA, 0.5 μM of each primer, 0.2 mM of deoxyribonucleotide triphosphate mix, (Invitrogen Carlsbad, CA, USA), 1.5 mM magnesium chloride, 1× buffer and 2.5 Unit Taq Polymerase (Invitrogen). The PCR conditions were as follows: denaturation at 94 °C for 3 min followed by 44 cycles of denaturation for 30 s, annealing at 58 °C–66 °C for 30 s, extension at 72 °C for 45 s, and final extension at 72 °C for 5 min. Bidirectional sequencing was carried out using the big dye terminator kit (Applied Biosystems, Foster City, CA, USA) on an automated DNA capillary sequencer (Model 3700; Applied Biosystems).

**Fig. 1:**
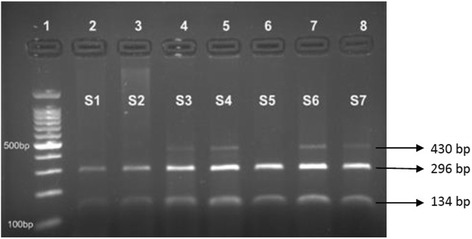
Restriction digestion of rs671 (ALDH2) PCR product: 430 bp using AcuI. Lane 1:100 bp ladder: Lanes 2–8: samples (S1–7); Lanes 4, 5, 7, 8: ALDH2*1/*2; Lanes 2, 3, 6: ALDH2*1/*1

**Fig. 2:**
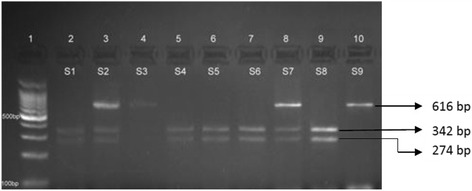
Restriction digestion of rs698 (*ADH1C*) PCR product: 616 bp using SspI. Lane 1: 100 bp ladder, Lane 2–10: samples (S1–9). Lanes 2, 5, 6, 7, 9: AA; Lanes 3, 4, 8: AG; Lane 10: GG

The rs671 of *ALDH2* gene was analysed using PCR and restriction fragment length polymorphism (RFLP). A 430-bp DNA fragment was amplified by PCR using the specific primers as per Helminen et al. 2013 [29]. The PCR protocol included, initial denaturation at 95 °C for 5 min followed by 44 cycles of 95 °C for 30 s, 60 °C for 30 s, and 72 °C for 45 s and a final extension at 74 °C for 5 min. PCR amplicons were digested using *Acu*I according to the manufacturer’s instructions (New England Biolabs Inc.). The 430 bp ALDH2*1 fragment was cut into two fragments of 296 and 134 bp and the ALDH2*2 allele (2*/2*) was not cut. Fragments were separated and analyzed by 2% agarose gel electrophoresis (Fig. 1). The rs698 of *ADH1C* gene was analysed using direct PCR amplification of 616 bp DNA fragment followed by *Ssp*I restriction digestion. The PCR protocol included one cycle of 94 °C for 5 min, 40 cycles of 94 °C for 30 s, 64 °C for 30 s, and 72 °C for 45 s and a final cycle of 74 °C for 5 min. PCR products were digested according to the manufacturer’s instructions (New England Biolabs Inc.). The 616 bp product with A allele was cut into two fragments of 342 and 274 bp while the G allele was not cut. Fragments were separated and analyzed by 2.5% agarose gel electrophoresis (Fig. 2). Samples of five randomly selected subjects were analyzed twice to assess the consistency of the genotyping protocol.

### *Helicobacter pylori* detection

*Helicobacter pylori* infection was detected in GC and control individuals by multiplex PCR amplification of 16S rRNA and CagA genes using specific primers [30]. The PCR amplification was carried out for 35 cycles at 95 °C for 45 s, 56 °C for 45 s, 72 °C for 1 min followed by a final extension at 72 °C for 10 min. Amplified PCR products were electrophoresed with 1.5% agarose gel. *Helicobacter pylori* infection was confirmed by the presence of an intact band of 109 bp (16S rRNA) and 400 bp (CagA gene).

### Statistical analysis

The genotypic data of each SNP were analysed by using multivariate logistic regression model. The t-tests (for continues variables) and chi-square tests (for categorical variables) were performed to compare the demographic variables and life style habits (smoking and alcohol consumption) between cases and controls. Hardy- Weinberg equilibrium of each SNP was examined using a χ2 test. Next, unconditional logistic regression model was used to evaluate the risk of gastric cancer with regard to smoking and alcohol status. All the tests were done using GraphPad InStat software (GraphPad InStat software, San Diego, CA) and SNPassoc version 1.8–1 software (Catalan Institute of Oncology, Barcelona, Spain). All *p*-values were adjusted for multiple comparisons using the False Discovery Rate (FDR) by Benjamini and Hochberg [31]. Linkage disequilibrium (LD) pattern was analyzed using Haploview 4.2. Survival curves were obtained according to Kaplan –Meier model. Overall survival was measured from the date of surgery to the date of most recent follow up or death (up to 2 years). SPSS 16.0 was used to perform this test. Power was estimated using Genetic Power Calculator.

## Results

### Characteristics of study participants

The basal characteristics and clinical data of the subjects are presented in Table 1. The mean ± SD age of patients was 55.43 ± 10.86 years (range 22–80 years) and 78% of them were males and 22% were females. There was a high frequency of occurrence of GC among males than that of females. Cases and controls appeared to be adequately matched with respect to age and gender as suggested by the chi square tests (*p* = 0.169 and 0.429 respectively, Table 1). The mean ± SD of BMI was 20.55 ± 2.775 kg/m2 in patients. In this study, we found 38% GC patients were underweight and no patients were identified with obesity. By anatomical location, we found 102 (98%) patients to be of non- cardia and only 3 (2%) were of cardia type. Histologically the sample population showed 49% intestinal, 23% diffuse and 28% indeterminate type. Significantly higher number of smokers (*p* = 0.001) and alcoholics (*p* = 0.001) were observed in cases compared to the controls (Table 1). Smokers had almost 2-fold increased risk of GC (OR = 2.45, 95% CI = 1.41–4.26, *p* = 0.001) and the use of alcohol also increased GC risk by 2-fold (OR = 2.77, 95% CI = 1.52–5.06, *p* = 0.001). This clearly indicates that smoking and alcohol had high risk burden for GC in our study population. Helicobacter pylori infection although was slightly higher in GC patients compared to controls but did not differ significantly between the two groups (Table 1). All patients included in our study were negative for family history.

**Table 1:** Basal characteristics and Clinical data of GC patients and controls

Characteristics	Control (*n* = 108)	Case (*n* = 105)	Odds ratio (95% CI)	*p* value
^a^Age (years ± SD)	53.64 ± 7.88 (range 20–80 years)	55.43 ± 10.86 (range 22–80 years)		0.169
Sex				
Male	89 (82.4%)	82 (78.0%)		
Female	19 (17.6%)	23 (22.0%)		0.429
^a^BMI (kg/m^2^)	23.28 ± 1.97	20.55 ± 2.75		<*0.001*
Anatomical location				
Cardia	–	3 (2.8%)		
Non-cardia	–	102 (97.2%)		
Histological subtypes of tumour				
Intestinal	–	52 (49.5%)		
Diffuse	–	24 (22.9%)		
Indeterminate	–	29 (27.6%)		
Alcohol consumption				
No	85 (78.7%)	60 (57.1%)		
Ever	23 (21.3%)	45 (42.9%)	2.77 (1.52–5.06)	*0.001*
Cigarette/bidi smoking				
No	66 (61.1%)	41 (39.0%)		
Ever	42 (38.9%)	64 (61.0%)	2.45 (1.41–4.26)	*0.001*
*Helicobacter pylori* positive	19 (17.6)	22 (21.0%)	1.24 (0.63–2.46)	0.534

^a^At diagnosis, *p* value < 0.05 is considered to be statistically significant

In our study, we found that weight loss (72%) was the commonest symptom followed by abdominal pain (68%), nausea/vomiting (58%), postprandial pain (47%), diarrhoea (42%) and malena (35%).

### *ADH* (*ADH1* gene cluster: *ADH1A, ADH1B* and *ADH1C*) and *ALDH2* gene polymorphisms

We investigated polymorphisms of *ADH1A* (rs1230025), *ADH1B* (rs3811802, rs1229982, rs1229984, rs6413413, rs4147536, rs2066702 and rs17033), *ADH1C* (rs698) and *ALDH2* (rs886205, rs671, rs968529 and rs16941667) genes (Additional file 3: Data S2), of which two SNPs (rs6413413 and rs2066702) of *ADH1B* showed monomorphic nature in our study population. The genotype distributions of rest of the SNPs were in Hardy-Weinberg equilibrium.

**Fig. 3:**
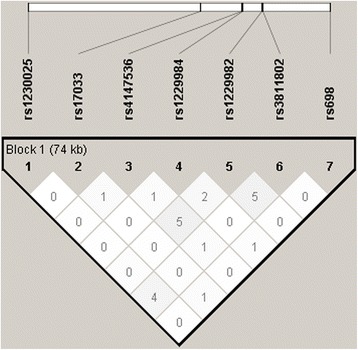
Linkage disequilibrium (LD) pattern (r2) of the seven SNPs in ADH1A, ADH1B and ADH1C gene. LD pattern of rs1230025 in ADH1A, rs17033, rs4147536, rs1229984, rs1229982 in ADH1B and rs698 in ADH1C gene in case and control groups. The LD between the SNPs is measured as r2 and shown in the diamond at the intersection of the diagonals from each SNP. r2 = 0 is shown as white, 0 < r2 < 1 is shown in gray and r2 = 1 is shown in black

We found that rs17033 and rs4147536 of *ADH1B* were associated with GC. The genotype and allele frequencies of these polymorphisms are given in Table 2. No linkage disequilibrium was observed among the 9 SNPs (Fig. 3).

**Table 2:** Genotype and allele frequencies of *ADH1A*, *ADH1B, ADH1C* and *ALDH2* gene and association with gastric cancer risk

Genotype	Controls(n-108) n (%)	Cases (n-105) n (%)	OR^a^ (95% CI)	*p*-value
*ADH1A* rs1230025				
TT	52 (48.1)	45 (42.9)	1.00	
TA	46 (42.6)	57 (54.3)	1.10 (0.55–2.19)	
AA	10 (9.3)	3 (2.9)	0.28 (0.05–1.55)	0.284
TT	52 (48.1)	45 (42.9)	1.00	
TA + AA	56 (51.9)	60 (57.1)	0.96 (0.49–1.87)	0.893
TT + TA	98 (90.7)	102 (97.1)	1.00	
AA	10 (9.3)	3 (2.9)	0.27 (0.05–1.44)	0.099
T allele	69%	70%	1.00	
A allele	31%	30%	0.95 (0.63–1.44)	0.819
*ADH1B* rs3811802				
TT	62 (57.4)	54 (51.4)	1.00	
TC	44 (40.7)	51 (48.6)	1.37 (0.79–2.38)	0.162
CC	2 (1.9)	0 (0.0)	–	
TT	62 (57.4)	54 (51.4)	1.00	
CT + TT	46 (42.6)	51 (48.6)	1.32 (0.76–2.29)	0.316
T allele	78%	76%	1.00	
C allele	22%	24%	1.09 (0.70–1.72)	0.697
*ADH1B* rs1229982				
CC	78 (72.2)	74 (70.5)	1.00	
CA	28 (25.9)	30 (28.6)	0.92 (0.42–2.01)	
AA	2 (1.9)	1 (1.0)	0.89 (0.06–13.66)	0.974
CC	78 (72.2)	74 (70.5)	1.00	
CA + AA	30 (27.8)	31 (29.5)	0.91 (0.42–1.99)	0.820
CC + CA	106 (98.1)	104 (99.0)	1.00	
AA	2 (1.9)	1 (1.0)	0.93 (0.06–13.87)	0.957
C allele	85%	85%	1.00	
A allele	15%	15%	1.00 (0.59–1.69)	0.991
*ADH1B* rs1229984				
GG	107 (99.1)	104 (99.0)	1.00	
GA	1 (0.9)	1 (1.0)	1.44 (0.02–130.3)	0.874
G allele	100%	100%	1.00	
A allele	0%	0%	–	–
*ADH1B* rs4147536				
GG	56 (51.9)	62 (59.0)	1.00	
GT	41 (38.0)	41 (39.0)	1.03 (0.53–2.00)	
TT	11 (10.2)	2 (1.9)	0.22 (0.04–1.12)	0.114
GG	56 (51.9)	62 (59.0)	1.00	
GT + TT	52 (48.1)	43 (41.0)	0.86 (0.46–1.62)	0.636
GG + GT	97 (89.8)	103 (98.1)	1.00	
TT	11 (10.2)	2 (1.9)	0.18 (0.04–0.82)	*0.009*
G allele	71%	79%	1.00	
T allele	29%	21%	0.66 (0.43–1.03)	0.066
*ADH1B* rs17033				
AA	101 (93.5)	85 (81.0)	1.00	
AG	7 (6.5)	17 (16.2)	2.38 (0.84–6.75)	0.054
GG	0 (0.0)	3 (2.9)	–	
AA	101 (93.5)	85 (81.0)	1.00	
AG + GG	7 (6.5)	20 (19.0)	2.80 (1.02–7.70)	*0.039*
A allele	97%	89%	1.00	
G allele	3%	11%	3.67 (1.54–8.75)	*0.002*
*ADH1C* rs698				
AA	60 (55.6)	61 (58.1)	1.00	
AG	41 (38.0)	34 (32.4)	0.62 (0.30–1.29)	
GG	7 (6.5)	10 (9.5)	2.04 (0.53–7.89)	0.189
AA	60 (55.6)	61 (58.1)	1.00	
AG + GG	48 (44.4)	44 (41.9)	0.76 (0.39–1.51)	0.435
AA + AG	101 (93.5)	95 (90.5)	1.00	
GG	7 (6.5)	10 (9.5)	2.40 (0.63–9.10)	0.196
A allele	75%	74%	1.00	
G allele	25%	26%	1.06 (0.69–1.65)	0.778
*ALDH2* rs886205				
AA	35 (32.4)	35 (33.3)	1.00	
AG	56 (51.9)	45 (42.9)	0.75 (0.34–1.63)	
GG	17 (15.7)	25 (23.8)	1.58 (0.59–4.21)	0.255
AA	35 (32.4)	35 (33.3)	1.00	
AG + GG	73 (67.6)	70 (66.7)	0.92 (0.44–1.93)	0.832
AA + AG	91 (84.3)	80 (76.2)	1.00	
GG	17 (15.7)	25 (23.8)	1.89 (0.81–4.43)	0.137
A allele	58%	55%	1.00	
G allele	42%	45%	1.13 (0.77–1.66)	0.518
*ALDH2* rs671				
GG	104 (96.3)	88 (83.8)	1.00	
GA	4 (3.7)	15 (14.3)	5.04 (1.37–18.57)	*0.021*
AA	0 (0.0)	2 (1.9)		
GG	104 (96.3)	88 (83.8)	1.00	
GA + AA	4 (3.7)	17 (16.2)	5.30 (1.46–19.20)	*0.006*
G allele	98%	91%	1.00	
A allele	2%	9%	4.20 (1.54–11.46)	*0.003*
*ALDH2* rs968529				
CC	101 (93.5)	101 (96.2)	1.00	
CT	7 (6.5)	4 (3.8)	0.36 (0.08–1.73)	0.371
C allele	97%	98%	1.00	
T allele	3%	2%	0.73 (0.23–2.33)	0.592
*ALDH2* rs16941667				
CC	104 (96.3)	97 (92.4)	1.00	
CT	3 (2.8)	7 (6.7)	3.49 (0.52–23.24)	0.294
TT	1 (0.9)	1 (1.0)	0.19 (0.00–11.13)	
CC	104 (96.3)	97 (92.4)	1.00	
CT + TT	4 (3.7)	8 (7.6)	2.03 (0.39–10.60)	0.395
CC + CT	107 (99.1)	104 (99.0)	1.00	
TT	1 (0.9)	1 (1.0)	0.19 (0.00–10.94)	0.408
C allele	98%	96%	1.00	
T allele	2%	4%	1.89 (0.62–5.73)	0.254

^a^Odds ratio were adjusted for age, sex, BMI, alcohol and smoking status, *p* value < 0.05 is considered to be statistically significant

Our results suggest that for rs17033, G allele is the risk allele (G vs A: OR = 3.67, 95% CI = 1.54–8.75, *p* = 0.002) towards the development of GC. Simultaneously, when we combined the variant AG genotype with the GG genotype (i.e., AG + GG), assuming a dominant genetic model, a 3 fold increased risk was observed (AG + GG vs AA; OR = 2.80, 95% CI = 1.02–7.70; *p* = 0.039). Our findings also suggest that individuals having TT genotype of rs4147536 had significantly decreased risk of GC (OR = 0.18; 95% CI: 0.04–0.82; *p* = 0.009).

For *ALDH2*, out of the 4 SNPs studied, rs671 (p.Glu504Lys), a well characterized functional SNP, was found to be associated with GC risk and A allele appeared to be the risk allele (A vs G: OR = 4.20, 95% CI = 1.54–11.46, *p* = 0.003) for GC. In all genotypes combined, the dominant model (i.e., GA + AA) of this SNP showed significant association with GC: OR = 5.30, 95% CI = 1.46–19.20, *p* = 0.006 (Table 2).

However, after FDR adjustment, rs17033 and rs671 was not found to be significant in the dominant genetic model.

### Stratification analyses of *ADH1B* rs17033, rs4147536 and *ALDH2* rs671 polymorphisms and risk of gastric cancer

Stratification analyses were conducted to evaluate the effects of *ADH1B* and *ALDH2* genotypes with the risk of GC according to smoking status, alcohol-consumption status and BMI (Table 3). No significant association was observed between rs17033 and smoking and alcohol-consumption status. However, smokers having T allele of rs4147536 showed decreased risk of GC (OR = 0.41, 95% CI = 0.18–0.97; *p* = 0.041). On the other hand, smokers having the Lys allele of rs671 significantly had a 7-fold increased risk of GC (OR = 7.58, 95% CI = 1.34–42.78; *p* = 0.009) in our study. We also found that individuals who both smoke and consume alcohol, having the Lys allele significantly increased (10-fold) their risk of GC (OR = 10.90, 95% CI = 1.16–102.44; *p* = 0.010).

**Table 3:** Interaction between *ADH1B* rs17033, rs4147536, *ALDH2* rs671 polymorphisms, smoking, alcohol consumption and BMI in gastric cancer patients

	Exposure	Status	Genotypes	Control(n-108)	Case (n-105)	OR (95% CI)^a^	*P* value
*ADH1B* rs17033	Smoking	Non-smoker	AA	64	38	Reference:	
			AG + GG	2	3	3.84 (0.42–35.44)	0.223
		Smoker	AA	32	47	Reference:	
			AG + GG	5	17	1.25 (0.33–4.67)	0738
	Alcohol	Non-alcoholic	AA	82	57	Reference:	
			AG + GG	3	3	1.79 (0.27–11.68)	0542
		Alcoholic	AA	19	28	Reference:	
			AG + GG	4	17	1.63 (0.40–6.60)	0491
	Smoking + Alcohol	Both non-smoker and non-alcoholic	AA	64	35	Reference:	
			AG + GG	2	3	3.84 (0.42–35.44)	0.223
		Both smoker and alcoholic	AA	19	25	Reference:	
			AG + GG	4	17	1.63 (0.40–6.60)	0.491
	BMI	<22	AA	23	53	Reference:	
			AG + GG	4	14	1.79 (0.22–14.79)	0.587
*ALDH2* rs671	Smoking	Non-smoker	GG	64	40	Reference:	
			GA + AA	2	1	0.28 (0.01–5.83)	0.396
		Smoker	GG	40	48	Reference:	
			GA + AA	2	16	7.58 (1.34–42.78)	*0.009*
	Alcohol	Non-alcoholic	GG	82	57	Reference:	
			GA + AA	3	3	0.69 (0.08–5.62)	0.725
		Alcoholic	GG	22	33	Reference:	
			GA + AA	1	7	2.15 (0.20–23.50)	0.512
	Smoking +Alcohol	Both non-smoker and non-alcoholic	GG	64	37	Reference:	
			GA + AA	2	1	0.28 (0.01–5.83)	0.396
		Both smoker and alcoholic	GG	22	28	Reference:	
			GA + AA	1	14	10.90 (1.16–102.44)	*0.010*
	BMI	<22	GG	25	56	Reference:	
			GA + AA	2	11	1.38 (0.14–14.01)	0.787
*ADH1B* rs4147536	Smoking	Non-smoker	GG	36	16	Reference:	
			GT + TT	30	25	1.75 (0.65–4.72)	0262
		Smoker	GG	20	46	Reference:	
			GT + TT	22	18	0.41 (0.18–0.97)	*0.041*
	Alcohol	Non-alcoholic	GG	45	33	Reference:	
			GT + TT	40	27	0.62 (0.17–2.20)	0.456
		Alcoholic	GG	11	29	Reference:	
			GT + TT	12	16	0.36 (0.08–1.69)	0.188
	Smoking +Alcohol	Both non-smoker and non-alcoholic	GG	36	16	Reference:	
			GT + TT	30	22	1.75 (0.65–4.72)	0.262
		Both smoker and alcoholic	GG	11	29	Reference:	
			GT + TT	12	13	0.62 (0.17–2.20)	0.456
	BMI	<22 Kg/m^2^	GG	17	41	Reference:	
			GT + TT	10	26	2.81 (0.62–12.62)	0.166

^a^Odds ratio were adjusted for age, sex, BMI, alcohol and smoking status, *p* value < 0.05 is considered to be statistically significant

### Combined effect of rs698 and rs671 polymorphism with GC risk

To elucidate the combined effect of both the polymorphisms, we considered individuals carrying both the minor alleles (G of rs698 and A of rs671) and compared them with individuals carrying either a single or no risk allele. We found that individuals carrying both the risk alleles showed 5 fold increased risk (*p =* 0.013; Odds ratio = 5.66; 95% CI: 1.22–26.14) of GC compared to individuals carrying a single or no risk allele.

### Patient survivability with *ADH1B* rs17033, rs4147536 and *ALDH2* rs671 polymorphism

**Fig. 4:**
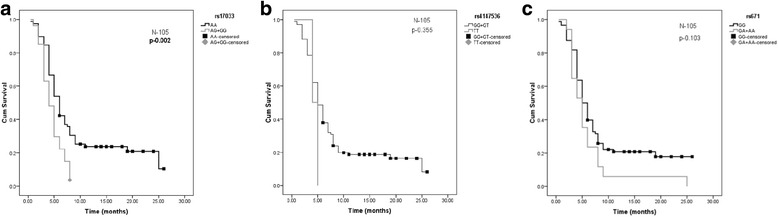
Kaplan-Meier 2-year survival probability curves with survival of GC patients by genotype status. **a** Survival probability curves with survival of GC patients by genotype status of rs17033 (AA vs GA + GG: *p* = 0.002). **b** Survival probability curves with survival of GC patients by genotype status of rs4147536 (GG + GT vs TT: *p* = 0.355). **c** Survival probability curves with survival of GC patients by genotype status of rs671 (GG vs GA + AA: *p* = 0.103)

The average survivals of all GC patients were 7.5 months and the median overall survival was 6 months. The mortality in GC patients with rs17033 risk genotype AG + GG was 92.3% versus 80.7% in the GC patients with non-risk genotype AA and Kaplan Meier survival analysis showed significant association between rs17033 and patient survivability (AG + GG vs AA: *p* = 0.002) (Fig. 4[a]). However, we did not find any association between rs4147536 (*p* = 0.355) and rs671 (*p* = 0.103) and overall survival (Fig. 4[b, c]).

## Discussion

GC is a multifactorial disorder developing from the inner lining of the stomach. It is mostly asymptomatic or present only non-specific symptoms in its early stages [2]. However, different studies have shown that abdominal pain, vomiting, dysphagia, weight loss and malena are the most predominant symptoms of gastric carcinoma [32, 33]. In our study, we found that weight loss was the commonest symptom followed by abdominal pain. Helicobacter pylori infection, though, is an established cause of GC, yet smoking, alcohol, diet, genetics and epigenetic factors may also play significant role in the occurrence of this disease.

Alcohol dehydrogenase, the rate limiting enzyme in alcohol metabolism, catalyzes the oxidation of ethanol to AA, which is then converted to acetate by aldehyde dehydrogenase. Genetic polymorphisms in the genes encoding both these enzymes have been associated to various cancers including tumors of the oral cavity, pharynx, larynx, esophagus and stomach [34]. There are only a few studies on the possible association between variants of *ADH1A*, *ADH1B*, *ADH1C* and *ALDH2* genes and GC. To date, one prospective study in Europe [20] and several case control studies [27, 35, 36] have reported associations between *ADH1A*, *ADH1B, ADH1C* and *ALDH2* polymorphisms and GC risk. Given the lack of reports linking these gene polymorphisms to GC in Asian populations, particularly Indian patients, this study sought to investigate the associations of *ADH1A* (rs1230025), *ADH1B* (rs3811802, rs1229982, rs1229984, rs6413413, rs4147536, rs2066702 and rs17033*), ADH1C (*rs698) and *ALDH2* (rs886205, rs968529, rs16941667 and rs671) SNPs with the risk of GC in a patient population from West Bengal, India.

A recent study has shown that rs1230025 (an intergenic SNP flanking the 3′ UTR of *ADH1A*) was associated with a 30% higher risk of GC in European population and the risk doubled when combined with *ALDH2* rs16941667 [20]. In contrast, we did not find any individual or combined influence of these SNPs on GC in our population. This difference in effect of these two SNPs may be due to the ethnic variation, life style and/or varied gene environmental interactions. Several polymorphisms have been identified in the *ADH1B* gene. Of note, rs1229984 and rs17033 have been considered to be important variants in the development of GC in Asian populations. The allele frequencies of rs17033 (T: 97%, C: 3%) in the present study were similar to that of South Asians (T: 96%, C: 4%), whereas the minor allele frequency was slightly different compared to Europeans (C: 9%) and Africans (C: 7%) **[**1000 genomes project]. In our study, multivariable logistic analyses revealed that the *ADH1B* rs17033 GG genotype (dominant model) was associated with GC risk. This, however, was found to be insignificant after FDR adjustment. Interestingly, the important functional polymorphism of *ADH1B*, rs1229984 was not associated with the disease in our study. On the other hand Asian populations, particularly the northeast Asians (i.e., Chinese, Japanese, and Korean), mainly harbor the ADH1B*47His allele (rs1229984 A). Similarly, in West Asian countries such as Iran and Turkey, where esophageal squamous cell carcinoma (ESCC) diagnoses are comparatively high, a corresponding high frequency of the ADH1B*47His allele is found. We detected one His (A) allele in our control group, the allele frequency was 0%, which is quite similar to South Asians (A: 2%) but differed significantly from East Asians (A: 70%) [1000 genomes project]. Therefore, geography and ethnic differences may be the probable reason behind the low frequency of rs1229984 polymorphism in our population as well as the lack of association with cancer risk. According to 1000 genomes project, the allele frequencies of rs698 in South Asians were A: 75%, G: 25%, which was quite similar to our result; however, the allele frequency was much different compared to East Asians and Europeans (A: 92%, G: 8% and A: 60%, G: 40% respectively). A meta-analysis performed on 35 case-control studies indicate that the*ADH1C* Ile350Val (rs698) polymorphism may contribute to cancer risk among Africans and Asians [23]. However, no association was observed between rs698 polymorphisms and GC risk in Japanese population [35]. We also observed no association of this SNP with GC further indicating that the role of individual alcohol dehydrogenase SNPs in increasing GC risk may be confined to specific ethnic populations.

A previous study has established the functional effect of the SNP rs1229982 in the proximal promoter region of *ADH1B* that was associated with alcoholism. They observed that a C to A change at rs1229982 increased the promoter activity by 1.4-fold [37]. This intergenic SNP although was not associated with GC risk overall, but was significantly associated with GC of the cardia in European population [20]. However, in our study we found no significant association of rs1229982 of *ADH1B* with GC. The rs6413413 and rs2066702 of *ADH1B* were monomorphic in our study population corroborating earlier findings in a Polish population [38]. In agreement with the results obtained in the 1000 genomes project for South and East Asian population, rs6413413 and rs2066702 of ADH1B were also monomorphic in our study population. *ADH1B* rs3811802 SNP, although polymorphic in our population, revealed no association with GC. Another intronic SNP, rs4147536 of *ADH1B,* might have a protective role in our study population. The minor allele (T) frequency of rs4147536 was 29%, which is exactly the same as South Asian population (T: 29%) [1000 genomes project]. Interestingly, smokers having the T allele of rs4147536 showed a decreased risk of GC (OR = 0.41, 95% CI = 0.18–0.97; *p* = 0.041). However, as no previous studies have linked the *ADH1B* SNPs rs3811802 and rs4147536 with GC risk, confirmation of a correlative link between these SNPs and GC warrants further study.

The major enzyme responsible for the elimination of AA is aldehyde dehydrogenase 2 [39]. Studies seeking to establish a link between *ALDH2* gene variants and GC have yielded conflicting results [35, 40]. A single-nucleotide alteration of *ALDH2*, the *ALDH2* *2 (504Lys: rs671 A) allele, results in a glutamic acid (glutamate) to lysine substitution at residue 504 rendering the protein inactive. Individuals harboring this mutation are unable to metabolize AA resulting in AA accumulation following alcohol intake [41]. Blood AA levels following alcohol consumption were 18 and 5 times higher in individuals homozygous and heterozygous for the ALDH2*2 variant, respectively [42]. Homozygous *2/*2 carriers, in particular, suffer severe acute AA toxicity exhibiting symptoms such as flushing, increased heart rate and nausea often precluding further alcohol intake. Heterozygotes, on the other hand, are still able to drink large amounts of alcohol despite increased AA accumulation. Previous studies have shown that the rs671 polymorphism was strongly associated with GC in an Asian population. In our study, *ALDH2* rs671 AA genotype (dominant model) was associated with an increased risk of GC consistent with the previous studies. However, after FDR adjustment, rs671 was not found to be significant in the dominant genetic model. While this allele is prevalent among East Asians (G: 83%, A: 17%) [1000 genome project]; *ALDH2* GA: 30–40%, *ALDH2* AA: 2.5–5% [43] and has not been detected in Caucasians or Africans [44], the genotype frequency was low in our population (3% for GA and 0% for AA). This inconsistency may due to small sample size, the unique population studied, dissimilar geographical areas and/or cancer type. Alcohol and tobacco smoke contains a number of carcinogenic substances that increase the risk of GC. In our study, investigation of gene –environment associations between genetic variations of *ALDH2* and drinking and smoking status indicated that rs671 and smoking synergistically increase risk of GC. We found that smokers having Lys allele of rs671 had a 7-fold increased risk of GC further validating previous reports [45]. In addition, individuals carrying both the rs698 and rs671 polymorphisms showed a 5 fold increased risk for GC compared to individuals carrying a single or no risk allele.

The link between cancer and another common functional variant in the *ALDH2* gene, rs886205, is also controversial. While a study on a Polish population reported that alcohol consuming individuals with the G allele had an increased risk of GC [38], Duell et al. [20], showed that rs886205 was not associated with GC risk overall but was significantly associated with GC of the intestinal subtype. Similarly, rs968529 and rs16941667 of *ALDH2* gene have been strongly linked to the intestinal subtype of GC [20], but a large meta-analysis has suggested that *ALDH2* rs886205 and rs16941667 might be strongly correlated with an increased risk of GC [46]. In our study, however, no positive relationships were found between these three SNPs of *ALDH2* (rs886205, rs968529 and rs16941667) and GC risk. The prognostic importance of the minor alleles of rs17033, rs4147536 and rs671 has been evaluated by Kaplan-Meier method. We found that the G allele of rs17033 was associated with the overall survival of GC patients.

The limitation of our study is the small sample size. In India, the incidence of gastric cancer (GC) varies across different registries. A higher incidence has been reported in the South compared to the North. The highest rate of GC cases is reported from the North Eastern state of Mizoram [47]. But the same is quite low in our state, West Bengal. As such, from December 1, 2012 to April 30, 2015, only 105 GC case samples were collected from IPGME & R, the only super specialty hospital in West Bengal.

## Conclusion

We conducted the first study regarding the associations between ADH1A, ADH1B, ADH1C and ALDH2 genes polymorphisms and the risk of GC from West Bengal, India. Our results indicate that rs17033 of ADH1B gene and rs671 of ALDH2 gene could be useful susceptibility molecular biomarkers for GC in our study population. Moreover, the combined effect of Glu504Lys (rs671) of ALDH2 with smoking significantly increases the risk of GC. In smokers, T allele of an intronic SNP, rs4147536 of ADH1B was shown to be associated with decreased risk of GC in our study population. Out results, though preliminary, suggest that it may be possible to identify genetic markers predisposing individuals to GC.

## Additional files

Additional file 1: Data S1. Gastric cancer patient report, Description of data- participant questionnaire used in the study. (DOCX 16 kb)

Additional file 2: Table S1. Primers using for amplification of SNPs of ADH1A, ADH1B, ADH1C and ALDH2 gene, Description of data- list all primers used in the study. (DOCX 14 kb)

Additional file 3: Data S2. Description of data- raw data of all the participants in the study. (CSV 11 kb)

## Abbreviations

AA: Acetaldehyde
ADH: Alcohol dehydrogenase
ALDH: Aldehyde dehydrogenase
CI: Confidential Interval
ESCC: Esophageal squamous cell carcinoma
GC: Gastric cancer
GI: Gastrointestinal
IPGME & R: Institute of Post Graduate Medical Education & Research
LD: Linkage disequilibrium
OR: Odds ratio
UTR: Untranslated region
