# Supplementary material for: Polymorphisms in ADH1B and ALDH2 genes associated with the increased risk of gastric cancer in West Bengal, India
Source: BMC Cancer. 2017 Nov 22;17:782. doi: 10.1186/s12885-017-3713-7 (PMC5700676; doi:10.1186/s12885-017-3713-7)
Supplement: Supplementary file 1 — Gastric cancer patient report, Description of data- participant questionnaire used in the study. (DOCX 16 kb) [file 12885_2017_3713_MOESM1_ESM.docx]

**S1 Data. Gastric cancer patient report**

Serial No.:

1. Name:
2. Age: Blood Group:
3. Sex:
4. Address:
5. Occupation:
6. Religion:
7. Final Diagnosis:
8. Indication of operation:
9. Date of admission:
10. Stay in hospital:

11. Date of discharge:

12. Date of death:

CHIEF COMPLAINTS:

1. Constipation/ diarrhea:
2. Bleeding PR:
3. Loss of appetite/ weight:
4. Vomiting:
5. Abdominal distention:
6. Abdominal pain:
7. Weakness/fatigue:
8. Anorexia
9. Hematemesis/malena:

PAST HISTORY

1. Previous similar complaints:

2. Previous surgeries:

3. Previous other malignancy:

4. H/O radiation exposure:

PERSONAL HISTORY:

1. DM:

2. HT:

3. BA:

4. Smoking:

5. Alcohol consumption:

6. Tobacco:

1. Diet: a. Vegetarianb. non-vegetarian
2. Socio-economic status:
3. Menstrual history:

FAMILY HISTORY:

1. Similar complaints:
2. Surgery in family:
3. Other malignancy:

EXAMINATION:

1. Build:

2. Nutrition:

3. Pallor:

4. Jaundice:

5. Cyanosis:

6. Clubbing:

7. Edema:

8. BP:

9. RR:

10. Temperature:

11. Abdomen: a.organomegaly:

b. pain:

c. distention:

LOCAL EXAMINAYION:

1. Size:

2. Shape:

3. Consistency:

4. Surface:

INVESTIGATION

1. CT:

2. USG abdomen:

3. Blood:

4. H. pylori:

4. UGIE:

5. Biopsy report:

6. Operation done:
