# Supplementary material for: Polymorphisms in ADH1B and ALDH2 genes associated with the increased risk of gastric cancer in West Bengal, India
Source: BMC Cancer. 2017 Nov 22;17:782. doi: 10.1186/s12885-017-3713-7 (PMC5700676; doi:10.1186/s12885-017-3713-7)
Supplement: Supplementary file 2 — Primers using for amplification of SNPs of ADH1A, ADH1B, ADH1C and ALDH2 gene, Description of data- list all primers used in the study. (DOCX 14 kb) [file 12885_2017_3713_MOESM2_ESM.docx]

**Table S1. Primers using for amplification of SNPs of *ADH1A, ADH1B, ADH1C* and *ALDH2* gene.**

| **Gene name** | **SNP** | **Primer** | **Sequence** | **Amplicon length** |
| --- | --- | --- | --- | --- |
| *ADH1A* | rs1230025 | FORWARD | 5’ CACCTGAGGTCAGGAGTTAAAG 3’ | 364bp |
|  |  | REVERSE | 5’CTTAGTGGGAGTGCTTCTTCTC 3’ |  |
| *ADH1B* | rs1229982 | FORWARD | 5'CGACCTTGTCCTAGATCTCAAAG 3' | 613bp |
|  |  | REVERSE | 5'GGAGGCATTGTAGAGGAACAG 3' |  |
| *ADH1B* | rs3811802 | FORWARD | 5'AGAACAGTGCGTGTCCTAAC 3' | 487bp |
|  |  | REVERSE | 5'AACCTGGCTTTAGCCTTCTC 3' |  |
| *ADH1B* | rs1229984  rs6413413  rs4147536 | FORWARD | 5'CAAGAAGGGAAGGTAGAGAAGG 3' | 484bp |
|  |  | REVERSE | 5'CAGAGAGGGAAAGAGGAAACTC 3' |  |
| *ADH1B* | rs2066702  rs17033 | FORWARD | 5'GGAAATAGGGTAGCTGGGATCA 3' | 538bp |
|  |  | REVERSE | 5'CCCAGCATGTGTATGTTCAGG 3' |  |
| *ADH1C* | rs698 | FORWARD | 5'GCTCCTTAACTTTGGGCATAAC 3' | 616bp |
|  |  | REVERSE | 5'GCCTTCAGCAGAATAGGAAATG 3' |  |
| *ALDH2* | rs886205 | FORWARD | 5’ GTCTCATCTCCTGGCCTTTG 3’ | 428bp |
|  |  | REVERSE | 5’ GCTGCCTCTACCATTCCTC 3’ |  |
| *ALDH2* | rs671 | FORWARD | 5′-TCAAATTACAGGGTCAACTGCT-3′ | 430bp |
|  |  | REVERSE | 5′-GGCTGGGTCTTTACCCTCTC-3′ |  |
| *ALDH2* | rs968529 | FORWARD | 5' ATGTAGTGATGCGTGCCTATAC 3' | 680bp |
|  |  | REVERSE | 5' GGAGGTGGAGATTGCAGTAAG 3' |  |
| *ALDH2* | rs16941667 | FORWARD | 5' GAGGCAAATGGGTTGAGCTA 3' | 629bp |
|  |  | REVERSE | 5' GTCTGGATCTTCTGACCTTGTG 3' |  |
